# Sertraline plus deanxit to treat patients with depression and anxiety in chronic somatic diseases: a randomized controlled trial

**DOI:** 10.1186/s12888-015-0449-2

**Published:** 2015-04-14

**Authors:** Limin Wang, Zhuoyuan Zhong, Jingyang Hu, Xiaoming Rong, Jun Liu, Songhua Xiao, Zhonglin Liu

**Affiliations:** 1 Department of Neurology, Guangdong General Hospital, Guangdong Academy of Medical Sciences, Guangdong Neuroscience Institute, 510080 Guangzhou, Guangdong Province P.R. China; 2 Department of Neurology, Sun Yat-sen Memorial Hospital, Sun Yat-sen University, 510120 Guangzhou, China

**Keywords:** Sertraline, Deanxit, Depression and anxiety, Chronic somatic diseases

## Abstract

**Background:**

Patients in chronic somatic diseases are often accompanied with depression and anxiety, remission of which may be observed in the third or fourth week after applying common antidepressant medications. We investigate the efficacy and safety of sertraline plus deanxit on patients with depression and anxiety in chronic somatic diseases.

**Methods:**

75 Patients who met the criteria were randomly assigned to deanxit group or placebo group: sertraline (75 mg/day) plus deanxit (one piece/day) (N = 38), or sertraline (75 mg/day) plus placebo (one piece/day) (N = 37) for 2 weeks, both groups received sertraline (75 mg/day) in the following 2 weeks. Changes from baseline to day 4, day 8, day 15, and day 29 in Hamilton Rating Scale for Depression (HAM-D) and Hamilton Rating Scale for Anxiety (HAM-A) total scores were the efficacy measures. Adverse events were monitored and registered systematically during the trial.

**Results:**

Response rates for HAM-D scores in deanxit group and placebo group were significantly different on day 8(55.26% ± 2.56% VS 24.32% ± 2.19%, p = 0.006) and day 15(78.95% ± 3.89% VS 40.54% ± 4.18%, p = 0.001), while no statistical differences were observed on day 4 and day 29. Respectively, response rates for HAM-A scores on day 4 (34.21% ± 2.21% VS 8.11% ± 1.37%, p = 0.006), day 8 (57.89% ± 3.56% VS 18.92% ± 2.68%, p = 0.001) and day 15 (78.95% ± 4.37% VS 43.24% ± 4.68%, p = 0.002), favoring the deanxit group. However, HAM-A scores were not remarkably different at the end point. The overall safety profile of both groups was favorable with no distinct differences.

**Conclusions:**

The efficacy was exhibited in the deanxit group, with evidence for similar safety. The rapid onset of sertraline plus short-term deanxit indicated that it might be an inspiring strategy to manage depression and anxiety within the first two weeks in chronic somatic diseases.

**Electronic supplementary material:**

The online version of this article (doi:10.1186/s12888-015-0449-2) contains supplementary material, which is available to authorized users.

## Background

Patients are notably affected by complications in chronic diseases predominantly mediated by social determinants of health, suffering discomfort for a long time [1,2]. Depression and anxiety are highly prevalent in persons with chronic somatic diseases such as diabetes, hypertension, heart disease, chronic bronchitis, neurological diseases, and significantly associate with physical health [3]. Similarly, individuals with depression and anxiety tend to at higher morbidity risks of the above mentioned chronic physical conditions [4]. Mood disorders exert a substantial impact on the health-related quality of life, the functioning and mortality of persons with chronic somatic diseases [5-7]. The comorbidity of depression and anxiety disorders is commonly unrecognized and untreated [7], as a result of which leads to somatic symptoms exacerbating [7,8], complications worsened [9] as well as compliance weakened [10]. With identification and medical management, patients suffering psychiatric disorders can have symptoms relieved, thus optimal health outcomes may be catalyzed [11].

Sertraline, an antidepressant of the selective serotonin reuptake inhibitors and serotonin-specific reuptake inhibitor (SSRIs) class, displays a rather beneficial balance between efficacy and acceptability in the acute-phase treatment of adults with major depression. Referring to a meta-analysis of 12 new-generation antidepressants, the cumulative probabilities of efficacy and acceptability of sertraline are 20.3% and 21.3% [12]. Randomized clinical trials have indicated that the sertraline is an efficacious treatment for anxiety and depressive disorders, along with low fatal toxicity in a good tolerability profile [13]. However, responses can be observed only 2 or 3 weeks later, even in a longer period of time when antidepressant drugs are prescribed [14]. Patients accompanied by depression and anxiety in chronic disease have poor compliance, even abandon treatments. Thus, there becomes an essential event to manage the symptoms of depression and anxiety as soon as possible, especially in the first two weeks.

Deanxit, a mixture of melitracen (10 mg) and flupentixol (0.5 mg), of which agents are accordingly a kind of tricyclic antidepressant and classical antipsychotic component, has been proven a rapid onset with both anxiolytic and antidepressant properties in low doses [15]. The biological half-life of flupentixol is about 35 hours and melitracen is about 19 hours, and the drugs show synergistic effect on therapeutic administration and antagonistic effect on adverse reaction. The combination of two psychoactive agents which has antidepressant properties is designed for short-term usage only. According to published evidences, melitracen/flupentixol combination is the most frequently prescribed compound on the basis of defined daily doses in China [16]. Deanxit can improve mood illness to some extent when prescribed in low dose for a short period of time, thus compensating the shortage of delayed response of sertraline. More generally, during the no response time for at least two weeks, deanxit is well tolerated.

Currently, consensus had been reached worldwide that early antidepressant treatments and timely eradication of the emotional disorder should come to realization imminently, therefore in which context, we designed a placebo-controlled study to assess the efficacy and safety of short-term combination of deanxit in the acute treatment on depression and anxiety in patients with chronic diseases. Basing on the therapeutic onset and tolerability, we employed a relatively conservative approach that prescription of deanxit only for the first 2 weeks in this study. To the best of our knowledge, controlled clinical studies in this design proposal were fairly rare.

As well as investigation of efficacy and safety of Deanxit, whether sertraline could acts rapidly was tested. We assumed that sertraline plus deanxit group would establish superiority versus sertraline plus placebo group in terms of efficiency and response rates in a short therapeutic period with equivalent safety.

## Methods

This 4-week, randomized, double-blind, placebo-controlled study was conducted from August 2011 to February 2014 at Sun Yat-sen Memorial Hospital of Sun Yat-sen University and Guangdong General Hospital. The project was registered by Chinese Clinical Trial Registry (Registration number: ChiCTR-TRC-11001732) on 25 November 2011.

### Patients

Patients were recruited from inpatients or outpatients in Guangdong General Hospital and Sun Yat-sen Memorial Hospital of Sun Yat-sen University. Patients either males or females who were aged from 20 to 75 years and suffered from chronic somatic diseases meeting the DSM-IV criteria for depression and anxiety were eligible for inclusion. Moreover, the intent-to-treat patients were required to have a score > 20 on HAMD and score >14 in HAMA at the time of screening. Patients were excluded if they suffered from acute diseases or severe mental illnesses that could confuse assessment of depression and anxiety. The other exclusion criteria included the history of epilepsy; the current treatment with antidepressants or antianxiety medications; the serious heart disease or serious hepatic disease or serious renal disease; language difficulties including dyslexia; pregnant or lactating women.

### Ethics

Ethical approval was obtained from each of the two sites. The project was approved by the Ethical Committee of Sun Yat-sen Memorial Hospital, Sun Yat-sen University, Guangzhou, China (approved number: 201005), and in accordance with the Helsinki Declaration of 1975. All patients provided written informed consent before entering the study.

### Study design

This study included 3 phases: a 1-week screening period; a 4-week, randomized, double-blind, placebo-controlled treatment period; followed by a 2-week follow-up period. Eligible patients were divided into two groups by randomization.

Patients received sertraline at a dosage of 75 mg/day plus deanxit (melitracen 10 mg + flupentixol 0.5 mg) or placebo at one piece every morning for 2 weeks, then both groups received only sertraline treatment at a dosage of 75 mg/day for the next 2 weeks. Tablets of placebo were identical in appearance compared with deanxit. After the end of the double-blind treatment period, the follow-up for 2 weeks were conducted in all patients.

This study medication was offered by the hospital pharmacy without signs of content. The allocation of treatment was managed by a centralized telerandomization system. Treatment compliance depended on retrieved tablet amounts at each interview. The compliance of < 90% defined as discontinuation. During the whole experimental process, patients were also taking matching medications for their chronic somatic diseases.

### Depression and anxiety severity assessments

The Hamilton Rating Scale for Depression (HAM-D) with 24 items (9 items were defined from 1 to 2, 1 item was defined from 0 to 2, and 14 items were defined from 0 to 4) was used for assessing the severity of depression, and the Hamilton Rating Scale for Anxiety (HAM-A) with 14 items (each item from 0 to 4) was applied for measuring the severity of anxiety.

The measurements of HAM-D and HAM-A were tested at baseline and before taking medication on day 1, day 4, day 8, day15, and day 29 in two groups. Scales were managed by two trained physicians.

### Treatment effect assessment

Score-reducing rate, expressed as (pre-treatment score of HAMD/HAMA – after-treatment score of HAMD/HAMA)/ pre-treatment score of HAMD/HAMA, was used to evaluate the treatment effect. According to clinical cure and improvement standards, the criteria of curative effect were as follow: score-reducing rate > 75% was recovery, 50 ~ 75% meant significant improvement, 25% ~ 49% stood for improvement and <25% as ineffectiveness. We defined response rate as the percentage of patients who had score-reducing rate ≥25% in the group.

### Safety and tolerability measures

The safety and tolerability of sertraline and deanxit were mainly detected by observing and reporting adverse events (AEs). Vital signs measurements (blood pressure, respiration rate, and pulse rate), cardiogram test and clinical laboratory tests (hematology, chemistry, and urinalysis) were used for additional safety reference.

### Statistical analysis

Two independent sample t-tests were performed to compare the difference between the characteristic of two groups. Chi square test was used to compare the difference of treatment efficiency. All analyses were conducted using SPSS version 16.0. Significance tests were 2-tailed and managed at the 0.05 significance level.

## Results

### Patient recruitment and allocation

**Figure 1:**
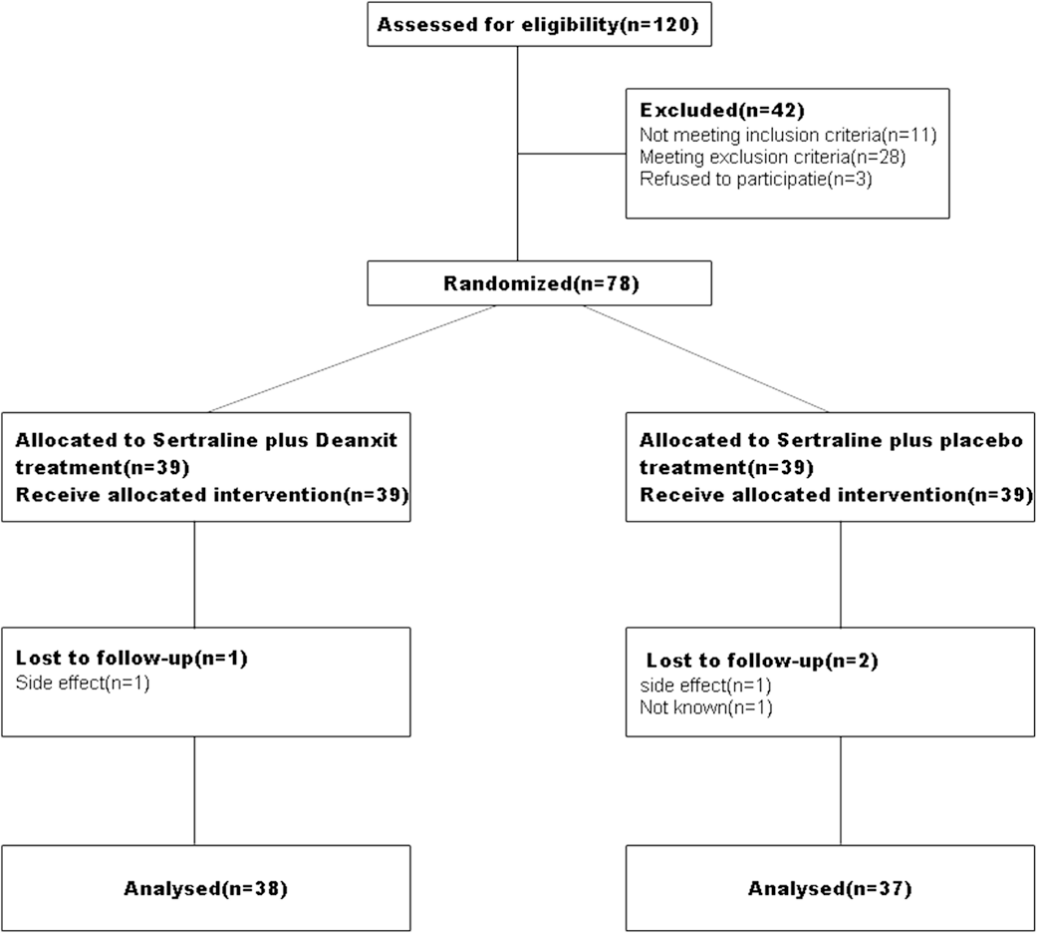
CONSORT flow chart of participants through each phase in the study.

As shown in Figure 1, recruitment and allocation of patients were summarized. 120 patients were assessed for eligibility; however, 42 patients were excluded from the study. 1 patient dropped out from deanxit group and 2 patients dropped out from placebo group. At last, only 75 patients were available for analyzed after the end phase of the study, of which 38 patients in deanxit group and 37 patients in placebo group.

### Patient disposition and characteristics

The baseline characteristics of the patients (n = 75) were outlined in Table 1. There were no significant differences in age, gender, BMI and employment status of the patients between deanxit group and placebo group.

**Table 1:** **Patients characteristic**

Variable	Sertraline plus deanxit	Sertraline plus placebo	p value
	(N = 38)	(N = 37)	
Age(yr)			
Mean ± SD	62.8 ± 13.1	61.5 ± 13.3	NS
Gender(%)			
Male	13(34.2%)	15(40.5%)	NS
Female	25(65.8%)	22(59.5%)	NS
BMI(body mass index)			
Mean ± SD	28.3 ± 4.1	28.0 ± 4.3	NS
Employment status			
Employed	14(36.8%)	12(32.4%)	NS
Unemployed	24(63.2%)	25(67.6%)	NS
Categories of chronic diseases			
Single hypertension	7	7	NS
Single diabetes	8	8	NS
Single chronic headache	6	5(droup out = 1)	NS
Single Parkinson’s disease	5(droup out = 1)	6	NS
chronic obstructive pulmonary diseases	5	5	NS
co-morbidity two or more chronic somatic disease	8	7(droup out = 1)	NS
Baseline score of HAMD			
Mean ± SD	31.6 ± 4.9	33.3 ± 5.1	NS
Baseline score of HAMA			
Mean ± SD	22.39 ± 4.3	23.0 ± 4.5	NS

Notes: NS = No Significant.

Baseline scores of HAMD and HAMA were tested to value the situation of depression and anxiety. Deanxit group showed 31.6 ± 4.9 score on HAMD and placebo group showed 33.3 ± 5.1 score on HAMD, which meant all the patients were in depression and the differences between the two groups were not significant (p > 0.05). The differences of baseline score on HAMA between deanxit group (22.39 ± 4.3) and placebo group (23.0 ± 4.5) also showed no significance (p > 0.05).

### Treatment effect of depression

**Figure 2:**
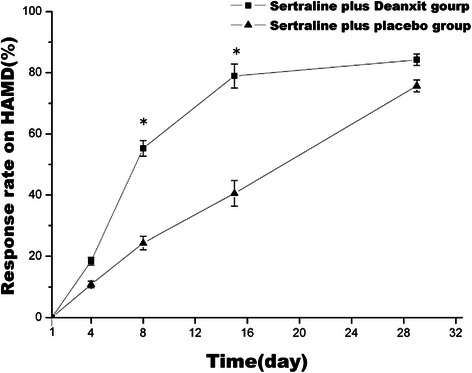
Change of total effective rate on depression from baseline in two groups. Date represented Mean ± SEM. * means P < 0.05.

As was shown in Figure 2, response rates on depression increased in both two groups as time went on. Response rates on HAMD in deanxit group on day 4, day 8, day 15, and day 29 were 18.42% ± 1.23%, 55.26% ± 2.56%, 78.95% ± 3.89%, and 84.21% ± 1.86%, and in placebo group were 10.81% ± 1.08%, 24.32% ± 2.19%, 40.54% ± 4.18%, and 75.68% ± 1.95%. The differences were significant on day 8 (p = 0.006) and day 15 (p = 0.001), whereas, the two groups displayed no statistically significant differences on day 4 and day 29 (p > 0.05).

### Treatment effect of anxiety

**Figure 3:**
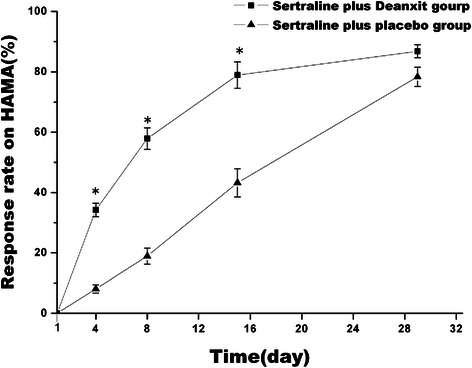
Change of total effective rate on anxiety from baseline in two groups. Date represents Mean ± SEM. * means P < 0.05.

As was shown in Figure 3, as the trial continued, response rates on HAMA of two groups both increased. Response rates on HAMA in deanxit group on day 4, day 8, day 15, and day 29 were 34.21% ± 2.21%, 57.89% ± 3.56%, 78.95% ± 4.37%, and 86.84% ± 2.12%, and in placebo group were 8.11% ± 1.37%, 18.92% ± 2.68%, 43.24% ± 4.68%, and 78.38% ± 3.16%. The differences were enormous on day 4 (p = 0.006), day 8 (p = 0.001) and day 15 (p = 0.002). However, the two groups showed no significant differences on day 29 (p > 0.05).

### Adverse reactions

Table 2 indicates that the total adverse event cases in deanxit group were 10 and in placebo group were 11. The two groups both had adverse events such as dry mouth, dizziness, sleeping disorder. However, only deanxit group observed 1 case of mild limb rest tremor. Most of these adverse events occurred in the first week of therapy, and disappeared within a week without any corresponding treatment.

**Table 2:** **Adverse events comparison between deanxit group and placebo group(%)**

Adverse events	Sertraline plus deanxit (n = 38)	Sertraline plus placebo (n = 37)
Dry mouth	3(7.9%)	2(5.4%)
Dizziness	2(5.3%)	3(8.1%)
Sleep disorder	1(2.6%)	2(5.4%)
Mild limb rest tremor	1(2.6%)	0(0%)
Dry mouth and Dizziness	3(7.9%)	2(5.4%)

## Discussion

This study assessed the efficiency and efficacy of deanxit used as an adjuvant for short duration combining a SSRIs agent. Response rates on depression and anxiety increased remarkably for patients receiving deanxit, indicating deanxit is superior versus placebo during the early treatment period, though at endpoint no prominent differences in aspect of efficacy between the two groups were observed. At the same time, the antidepressant effect of SSRIs agent was confirmed to some extent.

SSRIs achieved outstanding advantages in the pharmacological treatment of depression with a high selectivity of molecular target [17,18], and had become the most frequently prescribed class of antidepressant drugs in China [16]. Furthermore, attentions of clinicians should be drawn to the delay of two or three weeks before clinical onset, in case that clinical application of SSRIs would be weakened without notice of the response speed [19]. While aimed to achieve of optimal antidepressant-induced improvement of depressive symptoms, it was widely accepted that patients should spend a long time ranging from 6 to 12 weeks [20]. What was worse, depression often combined with other psychiatric disorders, in particularly anxiety disorder [21], leading to much poorer compliance of patients. Hence, it was crucial to manage both depression and anxiety as soon as possible in patients with chronic diseases within the first 2 to 3 weeks.

The results of the current randomized, double-blind study indicated that the sertraline plus deanxit had better efficacy in treating depression and anxiety symptoms at the first 2 weeks of the study period, compared with the sertraline plus placebo group, which might contribute greatly to clinical application for worthwhile notice. The antidepressant efficacy of sertraline in the current study was consistent with the previous studies [13,22]. Moreover, it has been reported that diabetes-related self-efficacy may be improved by sertraline in patients with T2DM [23] and the depression-free interval following recovery from major depression was prolonged by maintenance therapy with sertraline [24,25]. Sertraline, the antidepressant mechanism of which including altering the functional connectivity of the hypothalamus-anchored resting brain network [26], achieved superior effectiveness in Alzheimer patients on depressive, cognitive, and behavioral symptoms [25]. Newly, Sertraline has been investigated to exhibit a promising anti-inflammatory effect by increasing anti-inflammatory cytokine interleukin-10 (IL-10) while suppressing interleukin-6 (IL-6), tumor necrosis factor-alpha (TNF-α) [27,28]. Though, several chronic diseases link closely with systemic, low-grade chronic inflammation [29], sertraline may have therapeutic effect on some chronic diseases independent of antidepressant and in that case, studies in the association between sertraline and chronic somatic diseases will be warranted in future.

The effect of short term deanxit (melitracen 10 mg + flupentixol 0.5 mg) was viewed to make a contribution to the improvement during the early treatment period. Melitracen was a bipolar thymoleptic with activation properties, exerting effects on both depression and anxiety, which was similar to imipramine and amitriptyline. In addition, tolerability of melitracen was improved with a somewhat faster onset of action. Flupenthixol acting as antagonists at various dopamine (D1-D5), serotonin (5-HT2), adrenaline (α1), and histamine (H1) receptors, specifically antagonizes D1 and D2 receptors, thus typically playing an essential role of antipsychotic without affecting the muscarinic acetylcholine receptors [30]. Compared with amitriptyline, flupenthixol could significantly improve symptoms of patients with depression at low doses with a rapid onset of action [31,32]. According to a clinical trial aimed at short-term treatment of functional dyspepsia, patients without depression or anxiety received melitracen plus flupenthixol showed good clinical responses with favorable tolerance [15]. As flupentixol/melitracen was adjuvant for 2 weeks of treatment in patients with gastroesophageal reflux disease who had developed emotional disorder, both gastroesophageal-reflux symptoms and psychiatric symptoms were remarkably better than those in the monotherapy group [33]. The exact mechanism of a rapid onset of action and better effect on sertraline plus deanxit group was still unclear. Deanxit may act as a kind of potentiator of sertraline. Our results revealed that discontinuing deanxit 2 weeks later, the efficiency of sertraline plus deanxit group was still higher than control group, although not reaching statistical significance. Therefore, more researches should be done to verify whether the role of deanxit itself for improving depression and anxiety or the interaction with sertraline, result in that sertraline exerted its effect more quickly and efficaciously. In addition, the issue that whether the two drugs had combined effect should be investigated further.

Adverse events reported previously were mild in both groups. Sertraline plus deanxit were relatively safe and well tolerated. Patients received sertraline plus deanxit exhibited non-significantly higher rates, and there was one person existing mild limb rest tremor while symptoms vanished without relevant treatment. In the whole of safety profile, long-term use of deanxit might develop tardive dyskinesia and tardive akathisia, which should be highlighted to be aware of withdrawing deanxit gradually without delay, thus avoiding severe adverse reaction in movement [34].

The limitations of this study included the relatively small number of patients, the short duration of the treatment, and the single-centre nature of the study. This study recruited only five kinds of chronic diseases, which might not stand typically for the whole. What was more, as a result of the small samples, we could not divided eligible patients by stratified randomization according to various categories of chronic somatic diseases. In addition, almost all of patients with chronic diseases took corresponding medicine, which might interact with sertraline or deanxit and confuse the effects. The results of this 4-week trial could not generalize to longer periods of treatment, hence further large scale and cross-sectional study in rigorously designed would be warranted due to the limited subjects as well as a variety of chronic diseases.

## Conclusion

This controlled study demonstrated a noteworthy effect of sertraline plus short-term deanxit in the management of depression and anxiety in patients with chronic diseases within the first two weeks. It may be a potentially useful therapeutic strategy for treatment of mental illnesses when combining deanxit with sertraline in clinical practices.

## Supplementary material

An additional document shows a completed CONSORT checklist [see Additional file 1].

## Additional file

Additional file 1:
**An additional document shows a completed CONSORT checklist.**
